# Retraction: Circulating MicroRNAs as a Novel Class of Diagnostic Biomarkers in Gastrointestinal Tumors Detection: A Meta-Analysis Based on 42 Articles

**DOI:** 10.1371/journal.pone.0251146

**Published:** 2021-04-29

**Authors:** The PLOS ONE Editors

Following the publication of this article [1], the editorial team has become aware that the complete author list and institutional affiliations were changed during revisions of the manuscript, but the methodology, overall results, and conclusions remained the same. The corresponding author listed on the published version of this article [1] indicated that the original authors of the manuscript were unwilling to make the requested revisions and pay the publication fee; consequently, they arranged to transfer copyright from the authors listed on the original submission to the authors listed on the published article.

As per the PLOS authorship criteria in place at the time of submission of this article [1], in order to qualify for authorship authors should have contributed to the conception and design of the work, acquisition and/or analysis and interpretation of data, as well as drafting and/or revising of the article. Those who have contributed to the work, but do not qualify for authorship should be listed in the acknowledgements. The information received suggests the current authors do not meet the authorship criteria and concerns remain regarding compliance with *PLOS ONE* authorship policy.

In light of the concerns affecting the authorship of this article [1], the *PLOS ONE* Editors retract this article.

The authors did not respond to the retraction decision.
